# Serum Detection of Anti-thyroid Peroxidase and Anti-thyroglobulin Antibodies in Chinese Patients With Pemphigus Vulgaris and Pemphigus Foliaceus and Literature Review

**DOI:** 10.3389/fimmu.2021.653356

**Published:** 2021-03-16

**Authors:** He-Xiao Wang, Yang Yang, Jing-Yuan Hu, Li-Ming Zhang, Yun-Fei Cai, Hao Guo, Ting Xiao, Hong-Duo Chen, Xing-Hua Gao, Shuai Qiao

**Affiliations:** ^1^ NHC Key Laboratory of Immunodermatology, Ministry of Education Key Laboratory of Immunodermatology, National Joint Engineering Research Center for Diagnosis and Treatment of Immunologic Skin Diseases, Department of Dermatology, The First Hospital of China Medical University, Shenyang, China; ^2^ Department of Dermatology, The First Hospital of China Medical University, Shenyang, China

**Keywords:** anti-TPO antibody, anti-Tg antibody, pemphigus vulgaris, pemphigus foliaceus, meta-analysis, Chinese population

## Abstract

**Background:**

Pemphigus is a rare but life-threatening autoimmune skin disease characterized by blistering on skin and/or mucous membranes. The physiological process of blister formation involves IgG antibodies against the desmogleins (Dsgs) and desmocollins (Dscs). Additional autoAbs have also been suggested to mediate the disease heterogeneity, such as anti-thyroid peroxidase (anti-TPO) and antithyroglobulin (anti-Tg) antibodies, the essential culprits of the immune system in autoimmune thyroid diseases.

**Purpose:**

To investigate the levels and antibody positivity of anti-TPO and anti-Tg antibodies in pemphigus patients.

**Methods:**

Antibody positivity and levels of anti-TPO and anti-Tg antibodies in pemphigus patients as compared to healthy controls were examined. A meta-analysis was conducted by reviewing six similar studies.

**Results:**

98 Chinese pemphigus patients and 65 healthy controls were enrolled in the study. Our meta-analysis revealed a significant correlation between increased presence of positive anti-TPO and anti-Tg antibodies and pemphigus, particularly for pemphigus vulgaris (PV). Such correlation was also observed in our own hospitalized PV patients, but not in pemphigus foliaceus (PF) patients. In addition, the status of anti-TPO and anti-Tg antibodies were also compared between females and males within PV patients, PF patients or controls, as well as compared for females or males between pemphigus patients and controls. In the analysis of T cell counts, we found abnormal low CD3 + T cell counts (< 690 n/µl) were only detected in patients whose thyroid antibody levels were less than 20 IU/ml.

**Conclusion:**

Pemphigus patients showed higher levels and antibody positivity of anti-TPO and anti-Tg antibodies than healthy controls. Further investigations are needed to identify the pathogenic functions of these antibodies in pemphigus, as well as to identify the potential shared susceptibility genes.

## Introduction

Pemphigus is a rare but life-threatening autoimmune skin disease characterized by blistering on skin and/or mucous membranes (1, 2). The pathogenesis of pemphigus is rather complicated, with genetic and environmental factors being the most well recognized contributors to this severe disease (3). The physiological process of blister formation involves IgG antibodies against the desmoglein (Dsg) 1 and 3 (4, 5), and desmocollin (Dsc) 1-3 (6–8). The most common clinical variant of pemphigus, pemphigus vulgaris (PV), is generally associated with Dsg3 for the mucosal-dominant phenotype, and both Dsg1 and Dsg3 for the mucocutaneous/cutaneous phenotype (9). Whereas autoantibodies against Dsg1 are also present in patients with pemphigus foliaceus (PF), another major clinical variant of pemphigus that displays a phenotype of blistering in the superficial epidermis (10).

Although antibodies against desmosomal proteins are clearly correlated with disease progression in most pemphigus patients, however, accumulating evidence suggested the potential participation of additional autoAbs in determining the disease heterogeneity in pemphigus patients (11–14). Several studies have focused on antithyroglobulin (anti-Tg) and anti-thyroid peroxidase (anti-TPO) autoAbs (15–20). Correlation between PV and the presence of thyroid antibodies have been documented in these studies, although the role of thyroid antibodies in mediating the cell-cell adhesion remains unclear (21). Anti-TPO and anti-Tg antibodies are essential targets of the immune system in autoimmune thyroid diseases (AITDs), and increased prevalence of AITDs such as Hashimoto’s thyroiditis and Graves’s disease has been reported in pemphigus patients in several studies (22–24).

Here we examined the levels and positivity of anti-TPO and anti-Tg antibodies in 98 Chinese pemphigus patients and 65 healthy controls, and reviewed the available literature on association between increased presence of positive anti-TPO and anti-Tg antibodies and pemphigus, particularly for PV. Such correlation was also observed in our Chinese PV patients, but not in PF patients. A gender-based comparison on the status of anti-TPO and anti-Tg antibodies was also conducted in our PV and PF patients.

## Materials and Methods

### Electronic Search

Databases including PubMed, EMBASE, the Cochrane Library, China National Knowledge Infrastructure (CNKI), Wanfang database and Weipu database were systematically searched to identify all articles published to December of 2020. The search strategy consisted of the following index terms: (“Pemphigus Vulgaris” OR “Pemphigus Foliaceus” OR “Foliaceus, Pemphigus”) AND “Thyroid”. Additional manual search was also conducted on the references listed in identified articles.

### Inclusion and Exclusion Criteria

For inclusion, the studies needed to provide sufficient information to allow the analysis of antibody positivity for anti-TPO and anti-Tg antibodies in both pemphigus patients and control individuals. Studies enrolled patients with or without history of thyroid disease were both considered eligible. No restrictions on the methodologies for detecting levels of anti-TPO and anti-Tg antibodies were made. The studies were published in English or Chinese. Articles providing insufficient data, irrelevant to our topics, review article or case reports were excluded.

### Data Extraction and Quality Assessment

Two authors (H-X W, YY) independently evaluated all titles and abstracts of retrieved studies, and full texts that met the inclusion criteria were ultimately obtained. Disagreements on the eligibility of studies were resolved through discussion between the two authors, or if necessary, also with a third party (SQ). The following information was extracted from selected articles by two authors independently (H-XW, YY): name of the first author, publication year, country, sample size, type of pemphigus, thyroid function tests, gender, age and prevalence rate.

The quality of selected articles was assessed independently by two authors (H-XW, YY) by using the Newcastle-Ottawa-Scale (NOS). Each article was scored based on three dimensions: subject selection (0-3); comparability (0-4); and exposure (0-2). The total NOS score range from 0 to 8.

### Study Population

Information on 98 patients with a diagnosis of pemphigus (66 PV and 32 PF) at active phase was enrolled in our study. They were hospitalized patients of the Department of Dermatology at the First Hospital of China Medical University from 2013 to 2019. The study was approved by the ethics committee of the First Hospital of China Medical University (AF-SOP-07-1.1-01) and conducted according to the Declaration of Helsinki. Study participants gave written informed consent before they were included in the study. The diagnosis of pemphigus was confirmed based on clinical, histological, immunological (direct and indirect immunofluorescence) examinations and serological tests. Seventeen PV patients and ten PF patients were under corticosteroid therapy, immunoglobulin therapy or immunosuppressive therapy, while the other forty-nine PV patients and twenty-two PF patients were not under any treatment when the blood samples were drawn. Demographic, clinical and laboratory information of pemphigus patients were retrospectively collected from medical records. Control serum samples were collected from 65 healthy age- and sex-matched Chinese Han ethnic volunteers as described previously (25). Pemphigus patients with history of thyroid dysfunction or thyroid surgery, or malignancies were excluded.

### Chemiluminescence Microparticle Immunoassay

Evaluation of serum levels of anti-TPO and anti-Tg antibodies was performed according to the manufacturer’s protocols (Abbott Park, Middletown, USA). Levels of anti-Tg > 4.1100 IU/ml and anti-TPO > 5.6100 IU/ml were defined as antibody positive.

### ELISA

Serum samples were prepared at a dilution of 1:101, and each ELISA assay required 10 µl of undiluted serum sample. Elisa assay for anti-Dsg1 and anti-Dsg3 antibodies was performed using ELISA kit (MBL Intl, Japan) according to the manufacturers’ protocols. The detection range of anti-Dsg1 and anti-Dsg3 was 0-150 U/ml, and ELISA level > 20 U/ml was defined as Dsg antibody positive.

### T Cell Subset Counting (Flow Cytometry)

200 µL of peripheral blood were stained for markers of CD3, CD4 and CD8, and T cell subset counting was analyzed according the manufacturer’s instructions by flow cytometry instrument (BDCantoII). Normal counts for T cell subsets were: CD3: 690-2540 n/µl; CD4:410-1590 n/µl; CD8:190-1140 n/µl.

### Statistical Analysis

Statistical analysis of meta-analysis was performed by Stata software (version 15.0; Stata Corporation, Texas, USA). Pooled prevalence estimates and 95% confidence intervals (CIs) were calculated to determine the prevalence of positive thyroid antibodies in pemphigus patients. Sensitivity analysis was achieved by removing the study one by one to evaluate the reliability of the results.

Levels of anti-TPO and anti-Tg antibodies were summarized as means and were compared by the Mann-Whitney U test. Age, gender, prevalence of anti-TPO and anti-Tg antibodies between cases and controls, as well as prevalence of low T cell subsets between thyroid antibody positive group and negative group were compared using The Fisher’s exact test. Statistical analysis was performed by GraphPad Prism 7.0, and p-value < 0.05 was considered to be statistically significant.

## Results

### Study Selection and Characteristics

**Figure 1:**
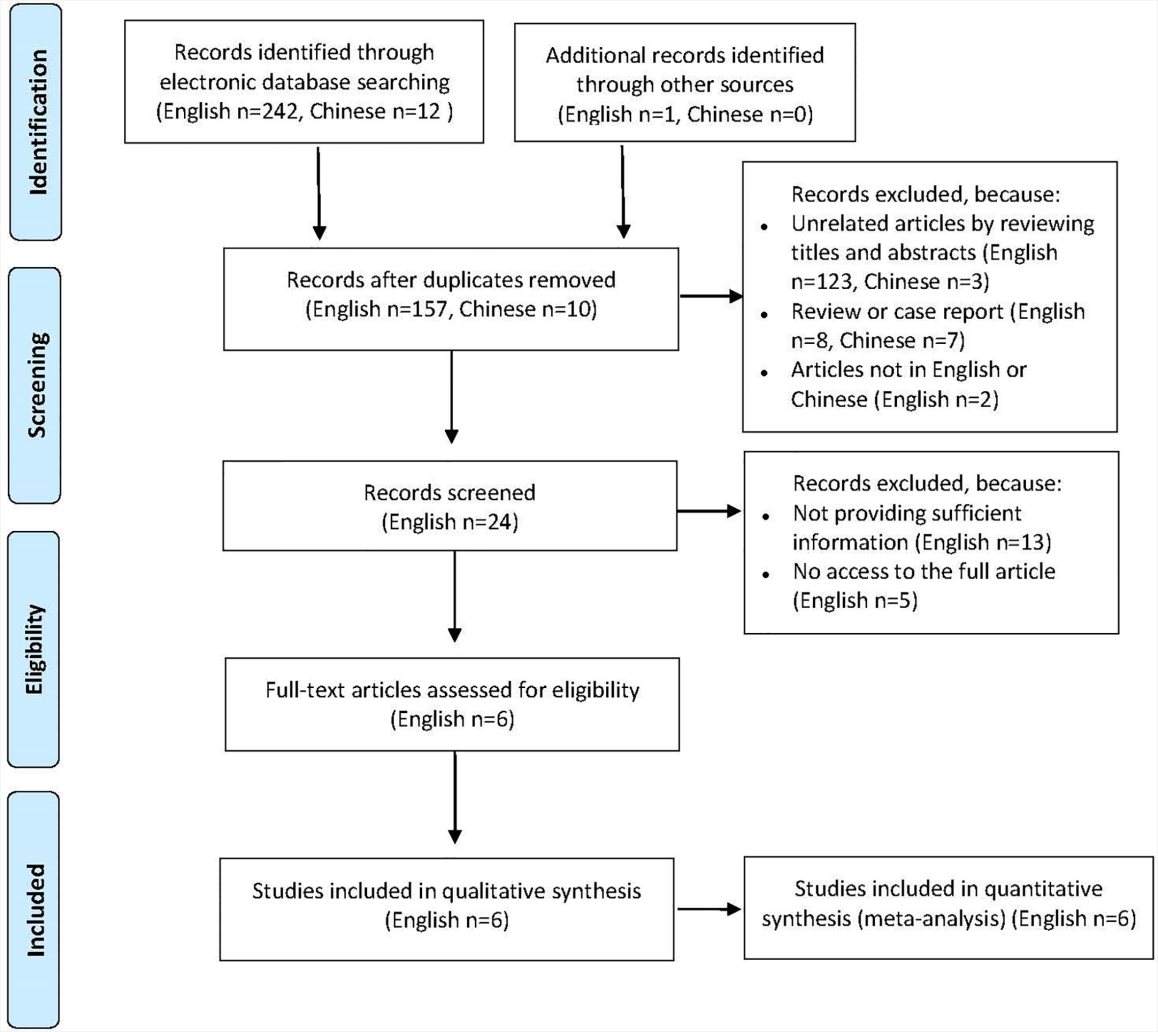
Flowchart of study selection process.

A total of 255 published articles were yielded based on the search strategy, and 6 articles were ultimately included in the meta-analysis after screening (**Figure 1**). Study characteristics and methodological quality were shown in **Table 1** (15–20). Six selected articles were published between 2005 and 2018. All studies included a patient group and a control group, and of which four were well matched on age and sex. The sample size of patient population varied between 15 and 225, and the patients reported in 6 studies were based in USA, Italy, Turkey, Iran and Argentina, respectively. All studies enrolled patients with PV and one study additionally enrolled patients with nonparaneoplastic pemphigus.

**Table 1:** Characteristics of studies on the prevalence of positive thyroid antibodies in patients with pemphigus.

Study	Country	Thyroid function tests	Pemphigus patients (PV)			Controls			Positive anti-TPO, %			Positive anti-Tg, %			NOS Score
			n	Female/Male	Mean Age (years)	n	Female/Male	Mean Age (years)	Patients	Controls	*p* value	Patients	Controls	*p* value	
Seiffert-Sinha et al. (15)	USA	Anti-Tg, Anti-TPO	225	148/77	52.9 ± 14.4	148	58/90	46.0 ± 18.7	13.9	7.2	0.042	6.8	0. 6	<0.001	7
Ameri et al. (16)	Italy	TSH, Anti-Tg, Anti-TPO	25	16/9	62.88 ± 16.54	46	20/26	63.26 ± 17.47	24	6.5	n.s.	12	2.2	n.s.	6
Kavala et al. (17)	Turkey	fT3, fT4, TSH, Anti-Tg, Anti-TPO	80	54/26	52.5	80	54/26	51.5	8	1.2	< 0.05	2.5	1.2	> 0.05	7
Daneshpazhooh et al. (18)	Iran	fT3, fT4, TSH, Anti-Tg, Anti-TPO	75	41/34	45.76 ± 1.79	65	28/37	41.13 ± 1.77	16	12.3	0.53	9.3	9.2	0.98	5
Ansar et al. (19)	Iran	T3, T4, TSH, Anti-TPO	22	14/8	35.04 ± 9.28	33	21/12	33.66 ± 9.13	22.7	6	< 0.05	n.a.	n.a.	n.a.	6
Pitoia et al. (20)	Argentina	T3, T4, TSH, Anti-TPO	15	9/6	48.3 ± 11.3	15	9/6	45.4 ± 12.2	40	6.7	n.a.	n.a.	n.a.	n.a.	7
Wang et al., the present study, 2021	China	Anti-Tg, Anti-TPO	66	36/30	51.65 ± 1.73	65	39/26	52.39 ± 0.75	16	6	0.034	20	12	0.1545	

n, Number; PV, pemphigus vulgaris; TG, thyroglobulin; TPO, thyroid peroxidase; TSH, thyroid stimulating hormone; T3, triiodothyronine; T4, thyroxine; NOS, Newcastle-Ottawa scale.

### Comparison of the Prevalence of Anti-TPO and Anti-Tg Antibodies in Pemphigus Patients and Controls

**Figure 2:**
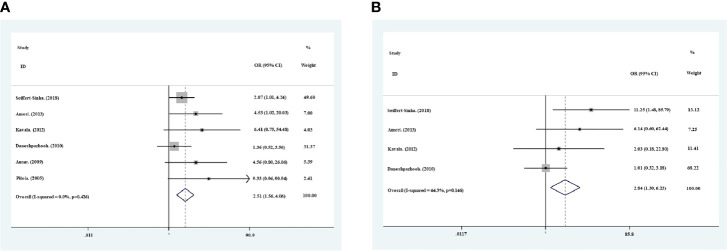
Meta−analysis using the fixed−effects model showed risk ratio of the prevalence of positive anti-TPO antibodies **(A)** and anti-Tg antibodies **(B)** in pemphigus patients compared with healthy controls.

The thyroid antibody profiles were reported in all 6 studies, with both anti-TPO and anti-Tg antibodies in 4 studies, and anti-TPO antibody only in 2 studies (**Table 1**). The prevalence of positive anti-TPO antibody in pemphigus patients varied between 8% and 40%, and that between 2.5% and 12% for anti-Tg antibody. Meta−analysis using the fixed−effects model suggested higher prevalence of positive anti-TPO (OR=2.51, 95% CI, 1.56−4.06) and anti-Tg (OR=2.84, 95% CI, 1.30−6.23) antibodies in patients with pemphigus than healthy individuals (**Figures 2A, B**). In addition, sensitivity analysis proved the reliability for the result of this meta-analysis.

### Detection of Anti-TPO and Anti-Tg Antibodies in Chinese PV Patients, PF Patients, and Healthy Controls

Based on the findings of higher frequency of positive anti-TPO and anti-Tg antibodies in pemphigus patients than controls by meta-analysis, we also detected the levels of anti-TPO and anti-Tg antibodies in 98 Chinese Han ethnic pemphigus patients (66 PV patients and 32 PF patients) and 65 healthy age-, sex-matched healthy controls (**Table 2**). In our investigations, by using the Fisher’s exact test we found PV patients have a significantly higher positivity of anti-TPO antibody than controls (24.24% in PV patients and 9.23% in controls, p<0.0001), but such difference was not observed between PF patients and controls (18.75% in PF patients, p=0.2017). The levels of anti-TPO antibody in PV patients (p<0.0001) and PF patients (p=0.0023) were also significantly higher than controls by Mann-Whitney U test. In the detection of anti-Tg antibody, we observed increased antibody level and positivity in both PV patients and PF patients, but with no statistical significance.

**Table 2:** Demographics of controls, Hashimoto’s thyroiditis (HT), PV patients and PF patients.

Characteristic	Controls (n=65)	Hashimoto’s disease (n=22)	PV (n=66)	PF (n=32)	p value (PV vs. Controls)	p value (PF vs. Controls)	p value (PV vs. HT)	p value (PF vs. HT)
Age, year (mean ± SEM)	52.39 ± 0.75	51.05 ± 1.78	51.65 ± 1.73	56.57 ± 2.03	0.86^	0.26^	0.808^	0.051^
Gender, female/male	39/26	11/11	36/30	18/14	0.597^	0.827^	0.807^	1.000^
Disease duration (months, mean ± SEM)			2.25 ± 0.25	6.54 ± 1.75				
Anti-TPO, IU/ml (mean ± SEM)	28.62 ± 16.24	470.95 ± 76.35	46.44 ± 21.71	28.70 ± 14.68	**<0.0001^#^**	**0.0023^#^**	<0.0001^#^	<0.0001^#^
Positive anti-TPO, n (%)	6 (9.23%)	22 (100%)	16 (24.24%)	6 (18.75%)	**0.034**^	0.2017^	<0.0001^	<0.0001^
Anti-Tg, IU/ml (mean ± SEM)	11.51 ± 5.43	209.05 ± 71.64	32.93 ± 12.31	57.00 ± 35.10	0.111**^#^**	0.282**^#^**	<0.0001**^#^**	<0.0001**^#^**
Positive anti-Tg, n (%)	12 (18.46%)	21 (95.45%)	20 (30.30%)	9 (28.13%)	0.1545^	0.3026^	<0.0001^	<0.0001^
Anti-Dsg1, U/ml (mean ± SEM)			116.06 ± 7.07	168.89 ± 19.31				
Anti-Dsg3, U/ml (mean ± SEM)			136.22 ± 8.17	3.15 ± 0.56				

PV, pemphigus vulgaris; PF, pemphigus foliaceus; TG, thyroglobulin; TPO, thyroid peroxidase.

Normal values: anti-TPO: 0-5.6100 IU/ml; anti-Tg: 0-4.1100 IU/ml; anti-Dsg1: 0-20 U/ml; anti-Dsg3: 0-20 U/ml.

^#^Mann-Whitney U-test.

^^^The Fisher’s exact test.

### Gender-Based Comparison of Anti-TPO or Anti-Tg Antibody Status in Chinese Controls, PV Patients, and PF Patients

**Figure 3:**
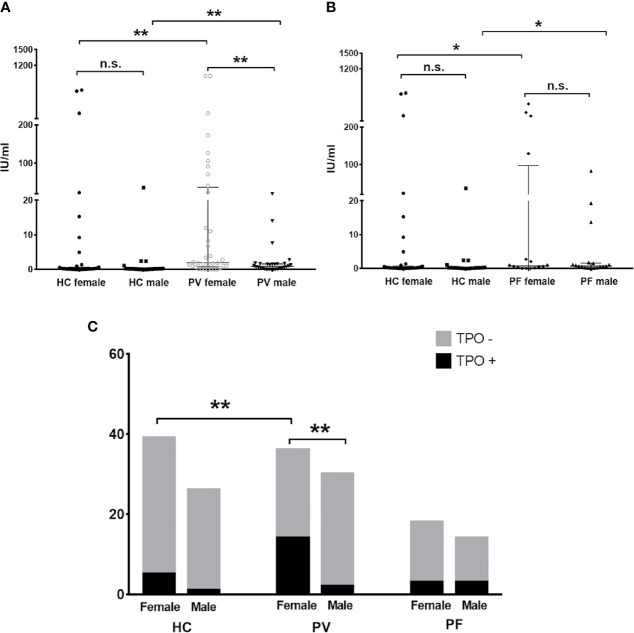
The status of anti-TPO antibody in PV patients, PF patients and healthy controls. **(A, B)** The levels of anti-TPO antibody in PV patients, PF patients and healthy controls, shown as median with interquartile range. **(C)** The antibody positivity of anti-TPO antibody in PV patients, PF patients and healthy controls. * indicates a significant difference (*p < 0.05, **p < 0.01, ***p < 0.001, by the Mann-Whitney U test for the comparison of antibody levels and The Fisher’s exact test for the comparison of antibody positivity).

The levels and antibody positivity of anti-TPO and anti-Tg antibodies were also compared between males and females within PV patients, PF patients or controls. As shown in **Figures 3A, C**, we found significantly higher levels and antibody positivity of anti-TPO antibody in PV female patients (83.34 ± 38.98 IU/ml, 38.89%) than PV male patients (2.16 ± 0.80 IU/ml, 6.67%) (p_level_ = 0.0081, p_positivity_ = 0.0032). In contrast to PV patients, the levels and antibody positivity of anti-TPO antibody in PF patients were likely to be higher in males (56.57 ± 32.13 IU/ml, 21.4%) than females (7.01 ± 4.63 IU/ml, 16.7%), although no statistical significance was reached (**Figures 3B, C**).

Moreover, we found the levels of anti-TPO antibody in female PV patients and male PV patients were even higher than that of female controls (46.54 ± 26.80 IU/ml, p=0.0013) and male controls (1.75 ± 4.10 IU/ml, p=0.0069), respectively. More female PV patients were also likely to carry anti-TPO antibody than female controls (12.82%) (p=0.0157). In PF patients, the levels of anti-TPO antibody were significantly increased in males and decreased in females when compared with male controls (p=0.016) and female controls (p=0.0278), respectively.

**Figure 4:**
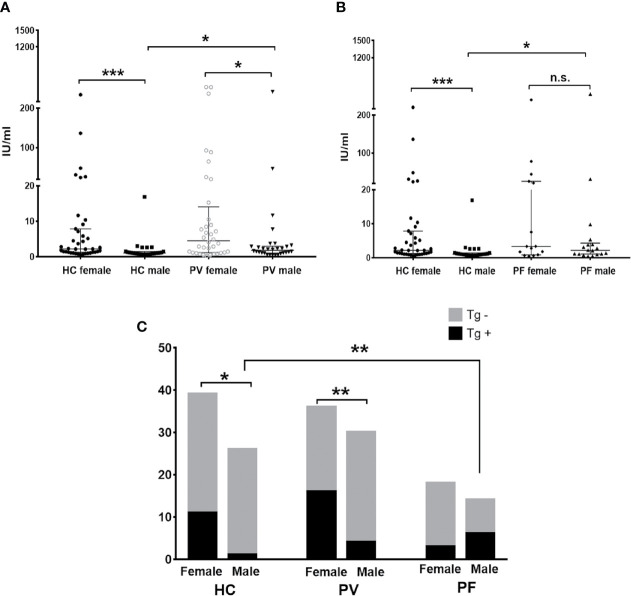
The status of anti-Tg antibody in PV patients, PF patients and healthy controls. **(A, B)** The levels of anti-Tg antibody in PV patients, PF patients and healthy controls, shown as median with interquartile range. **(C)** The antibody positivity of anti-Tg antibody in PV patients, PF patients and healthy controls. * indicates a significant difference (*p < 0.05, **p < 0.01, ***p < 0.001, by the Mann-Whitney U test for the comparison of antibody levels and The Fisher’s exact test for the comparison of antibody positivity).

For anti-Tg antibody detection, our results revealed significant differences in the levels and antibody positivity of anti-Tg antibodies between genders in both PV patient groups (females: 46.70 ± 6.28 IU/ml, 44.44%; males: 16.41 ± 12.63 IU/ml, 13.33%; p = 0.0286) and controls (females: 17.92 ± 8.95 IU/ml, 28.20%; males: 1.90 ± 0.61 IU/ml, 3.84%; p = 0.0003) (**Figures 4A, C**), but not in PF patients (females: 35.25 ± 31.01 IU/ml, 16.67%; males: 84.95 ± 70.64 IU/ml, 42.9%; p = 0.4860) (**Figures 4B, C**). For females or males between pemphigus patients and controls, however, differences in the levels or antibody positivity of anti-Tg antibodies were only detected in males between patients and controls (p_level_, PV/C = 0.0366, p_level_, PF/C = 0.0238, p_positivity_, PF/C = 0.0044).

### Abnormal Low CD3 + T Cell Counts Only Present in Chinese Pemphigus Patients With Low Levels of Thyroid Antibodies

The correlation between the absolute number of T cell subsets and thyroid antibodies in pemphigus patients were also investigated in 58 PV patients and 34 PF patients for whom the T cell subset counts in peripheral blood were available. It was very interesting to find abnormal low CD3 + T cell counts (< 690 n/µl) were only present in patients whose thyroid antibody levels were less than 20 IU/ml, although with no statistical significance (**Table 3**). These patients also showed abnormal low numbers of CD4 + T cells (< 410 n/µl) and/or CD8 + T cells (< 190 n/µl). No correlation between other T cell subsets and thyroid antibodies were observed.

**Table 3:** Prevalence of abnormal low T cell subset counts in the peripheral blood of PV patients and PF patients.

	PV Patients						PF Patients					
	anti-TPO +(n=9)	anti-TPO –(n=49)	p^^^	anti-Tg + (n=9)	anti-Tg - (n=49)	p^^^	anti-TPO + (n=4)	anti-TPO - (n=30)	p^	anti-Tg + (n=6)	anti-Tg - (n=28)	p^^^
n, % (CD3 count < 690 n/µl)	**0 (0)**	6 (12.24%)	0.576	**0 (0)**	6 (12.24%)	0.576	**0 (0)**	3 (10%)	> 0.999	**0 (0)**	3 (10%)	> 0.999
n, % (CD4 count< 410 n/µl)	1 (11.11%)	11 (22.45%)	0.668	2 (22.22%)	8 (16.32%)	0.646	1 (25%)	5 (16.67%)	0.559	2 (33.33%)	4 (14.29%)	0.620
n, % (CD8 count < 190 n/µl)	1 (11.11%)	7 (14.29%)	> 0.999	0	8 (16.32%)	0.334	1 (25%)	3 (10%)	0.431	1 (16.67%)	3 (10.71%)	> 0.999

n, number; PV, pemphigus vulgaris; PF, pemphigus foliaceus; TG, thyroglobulin; TPO, thyroid peroxidase.

Normal counts for T cell subsets: CD3: 690-2540 n/µl; CD4:410-1590 n/µl; CD8:190-1140 n/µl; Values > 20 IU/ml for anti-Tg or anti-TPO were defined as antibody cut-off.

^^^The Fisher’s exact test.

## Discussion

The present study has analyzed the antibody positivity and levels of anti-TPO and anti-Tg antibodies in pemphigus patients as compared to healthy controls, by performing a meta-analysis on 6 published studies, and also by examining our own hospitalized Chinese pemphigus patient population.

Additional autoimmune diseases can co-occur in individuals that are affected by an autoimmune disease, a concept known as ‘autoimmune diathesis’ (26, 27). This spurred the notion of investigating shared pathogenesis, as well as developing preventive and therapeutic strategies for these diseases (28). The most common autoimmune diseases, AITDs have been found to be co-occurred with certain autoimmune diseases, such as rheumatoid arthritis (RA), multiple sclerosis (MS), vitiligo and pemphigus (4).

Anti-TPO and anti-Tg antibodies are hallmarks of AITDs and elevated levels of these antibodies were even detectable several years before the clinical diagnosis of AITDS (29). Vitiligo patients were recommended to screen for these two antibodies due to their increased susceptibility in developing AITDS (30). Likewise, the correlations between pemphigus and anti-TPO/anti-Tg antibody positivity have been reported in several articles (15–20). We systematically searched relevant articles for a meta-analysis, and in result we identified PV patients tend to carry more anti-TPO and anti-Tg antibodies than healthy controls, implying PV patients may be at increased risk of developing AITDs than controls. In accordance with this finding, we have also confirmed higher prevalence of positive anti-TPO antibody in our own Chinese hospitalized PV as compared to healthy controls, but unfortunately not in PF patients. To our knowledge, this is the first study that investigated the thyroid antibody status in Chinese pemphigus patient population. PF patients were also rarely discussed for their thyroid antibody status, although only 32 cases of PF were included in this study due to the disease rarity. Significantly higher serum level of anti-TPO antibody was detected in our PV patients and PF patients as compared to controls, and the finding in PV patients was also consistent with two studies on Iranian and Turkish PV patients (17, 19). The levels and frequency of positive anti-Tg antibody were also likely to be higher than controls for both PV and PF patients, although with no statistical significance. These findings demonstrated generally consistent status of anti-TPO and anti-Tg antibodies between Chinese pemphigus population and previously reported other races.

It is well accepted that females are more susceptible to AITDs than males (31). Our PV female patients demonstrated higher levels and prevalence of anti-TPO and anti-Tg antibodies than males, and this finding was consistent with another study performed on American PV patients (15). In PF patients, however, both levels and antibody positivity of anti-TPO and anti-Tg antibodies were likely to be higher in males than females, although with no statistical significance, suggesting a potential higher tendency for PF male patients to develop AITDs and thyroid antibodies, and therefore, anti-TPO and anti-Tg antibodies should possibly be monitored particularly in PF male patients, and preventive strategies for AITDs may should also be performed. Whereas small sample size may also be an issue that contribute to the inconsistent results between PF patients and PV patients. Thus, validation of this finding should be performed on larger patient population and will be a feature of future studies by our group.

The impact of steroids on anti-thyroid antibodies was controversial. Ansar et al. implied autoimmune reactions could be suppressed in patients who received steroids (19), but Daneshpazhooh et al. found no significant difference in anti-thyroid antibodies between patients with and without steroid treatments (18). In accordance with Daneshpazhooh’ finding, steroids were also unlikely to influence the status of anti-thyroid antibodies in our PV and PF patients (data not shown), and this finding should be validated in the future study.

The presence of elevated anti-TPO and anti-Tg antibodies identified in pemphigus patients implied pemphigus and AITDs may potentially share susceptibility genes or alleles. Functions of influencing keratinocyte signalings by anti-TPO antibody may also be a potential explanation for the presence of elevated anti-TPO antibody in pemphigus (15).

In conclusion, our data has supported the potential correlation between thyroid antibodies and pemphigus. Particularly in Chinese population, significantly higher levels and positivity of anti-TPO antibody were observed in PV patients than controls. Further investigations are needed to be carried out to identify the pathogenic functions of anti-TPO and anti-Tg antibodies in pemphigus, as well as to identify the potential shared susceptibility genes. Given the impact on life quality and mortality of autoimmune disease, particularly for those with co-occurred conditions, quantification of anti-TPO and anti-Tg antibodies may be useful to identify pemphigus patients at risk for thyroid disease, and the necessity should also be verified in future studies on bigger sample size and more races.

## Data Availability Statement

The raw data supporting the conclusions of this article will be made available by the authors, without undue reservation.

## Ethics Statement

The studies involving human participants were reviewed and approved by the ethics committee of the First Hospital of China Medical University. The patients/participants provided their written informed consent to participate in this study.

## Author Contributions

H-XW analyzed and interpreted the data, and conducted the draft writing. SQ and X-HG contributed to the research concept and design, and draft revision. YY has contributed to literature searching and data extraction for the meta-analysis. J-YH and L-MZ have collected the serum samples and recorded patients’ medical information. TX and H-DC have revised the language and the content. HG has performed the serum testing. Y-FC has analyzed part of the data for meta-analysis. All authors contributed to the article and approved the submitted version.

## Funding 

This work was supported by NSFC (81903228 HG) and NSFC (81903198 QA) for data collection.

## Conflict of Interest

The authors declare that the research was conducted in the absence of any commercial or financial relationships that could be construed as a potential conflict of interest.
